# High-capacity and long-life CoO/C composite nanofibers as anode materials for lithium-ion batteries

**DOI:** 10.1039/d5ra07018j

**Published:** 2026-01-02

**Authors:** Zonghui Yi, Zhiyuan Cheng, Hui Zhang, Lin Xie, Haie Yang, Qian Du, Tao Wang

**Affiliations:** a College of Chemistry and Chemical Engineering, Ningxia Normal University Guyuan 756000 China zh0802@163.com +86-954-2079605 +86-954-2079607; b Ningxia Key Laboratory of Green Catalytic Materials and Technology, College of Chemistry and Chemical Engineering, Ningxia Normal University Guyuan 756099 China

## Abstract

Owing to its analogous conversion reaction mechanism and the same cobalt redox center with Co_3_O_4_, CoO was investigated as a high-capacity anode material for lithium-ion batteries in this study. The composite nanofibers, which were constructed by ultrafine CoO nanocrystals (∼5 nm) uniformly embedded in carbon matrix, were prepared by a simple electrospinning technique followed by high-temperature calcination. The resulting CoO/C composite nanofibers exhibited exceptional electrochemical performance. Notably, the CoO/C composite nanofiber prepared with 0.8 g of PVP (denoted as CoO/MC) delivered a charge capacity of 1147 mA h g^−1^ at 100 mA g^−1^ over 400 cycles. At a high current density of 1000 mA g^−1^, its charge capacity attained 578.7 mA h g^−1^, reaching 49.6% of that at 100 mA g^−1^. Kinetic analysis revealed that the diffusion-controlled contribution constituted a significant portion of the total capacity for the CoO/MC electrode. The excellent electrochemical performance of CoO/C composite nanofibers was related to inherent lithium storage property of CoO and their unique nanoarchitecture originated from a reasonable synthesis strategy. This work provides a new conceive to improve the lithium storage performance of metal oxide-based anode materials.

## Abstract 2

Owing to its analogous conversion reaction mechanism and the same cobalt redox center with Co_3_O_4_, CoO was investigated as a high-capacity anode material for lithium-ion batteries in this study.

## Graphical abstract

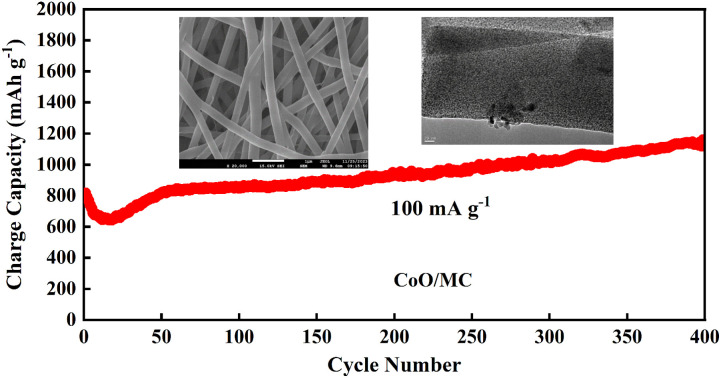

## 1. Introduction

Lithium metal, with a relative atomic mass of 6.941 g mol^−1^, exhibits the lowest density among all metals. The Li^+^/Li redox couple demonstrates the lowest standard electrode potential (*E*° = −3.04 V *vs.* SHE). Based on above-mentioned two issues, lithium-ion batteries (LIBs) allow storing much higher energy as compared to nickel-hydride or lead acid batteries with the same size. LIBs have progressively dominated the global electrochemical energy conversion/storage market in recent decades.^1–3^ However, expanding commercial applications of LIBs demand better performance in terms of energy and power densities. As a consequence, a continuous quest for electrode materials with improved energy and power densities is still underway. As an important component of LIBs, anode materials directly affect their energy density, cycle life, and safety.^4–6^ Graphite is currently the dominant anode material for LIBs due to its stable electrochemical performance and low cost. However, its intrinsic limitations in terms of low reversible capacity (a theoretical specific capacity of 372 mA h g^−1^) has gradually become a bottleneck for developing high-performance LIBs that demand both enhanced energy density and power density.^7–9^ In order to further upgrade the energy and power density of LIBs, alternative anode materials with higher specific capacity are urgently needed to further enhance LIB performance.^10–12^

Based on the charge storage mechanism, anode materials widely investigated for LIBs over the last decades can be broadly classified into three main types: intercalation reaction type, alloy reaction type, and conversion reaction type.^13–15^ Classical intercalation-type anode materials that retain their structure during electrochemical cycling, such as graphite and Li_4_Ti_5_O_12_, exhibit an intrinsic low capacity. This is caused by their structural aspects and redox mechanism.^16–18^ Alloy-type anode materials, such as Al and Si, deliver an extremely high capacity, but this extremely high capacity will cause huge volume changes and large strains in active particles during the (de)alloying process, resulting in a severe capacity fading.^19–23^ Additionally, most alloy-type anode materials consist of active metals or semimetals, making it difficult to synthesize them in nanosized dimensions and with pre-designed hierarchical structures. Most conversion-type anode materials are usually binary M–X compounds (M = transition metal, X = anion), including metal oxides, sulfides, phosphides, and selenides.^24,25^ During the discharge process, these conversion-type anode materials are reduced by Li to form corresponding M metals, which are embedded in the simultaneously generated Li_*n*_X matrix (*n* = formal oxidation state of X), such as Li_2_O or Li_2_S. During the charge process, M metals are oxidized by the Li_*n*_X to form binary M–X compounds again. Most conversion type anode materials were reported to provide stable gravimetric/volumetric capacities, significantly exceeding those of graphite. Among various conversion-type anode materials, binary 3d metal oxides deserve the most attention owing to their many interesting properties, such as relatively low atomic mass, high thermal stability, and microstructural tunability brought about their relatively inert nature. These binary 3d metal oxides exhibit reversible capacities far surpassing those of graphite-based anodes in LIB applications.^26–28^

Nonetheless, similar to other transition metal oxides, there are two intrinsic drawbacks that severely impair the electrochemical performance of binary 3d metal oxides. First, the electronic conductivity of 3d metal oxides is poor, resulting in their low actual capacity and unsatisfactory rate performance. Second, due to their high-capacity nature, a large number of Li^+^ ions insert/de-insert in/from active particles during the discharging/charging process, resulting in large volume changes in the binary 3d metal oxide particles. When the strains caused by such volume changes exceeds the tolerance limit of the oxide particles, these oxide particles will be pulverized. Some active particles eventually lose electrical contact with Cu current collector. As a consequence, their capacity is seriously attenuated. Generally speaking, the strategies conceived to circumvent aforementioned drawbacks include introducing conductive agents, designing nanostructured active particles, and constructing micro/nanostructured composites. Among these strategies, fabricating nanosized 3d metal oxide/conductive carbon composite is widely adopted, as the synergetic effect of nano-size effect and conductive carbon can significantly improve the electrochemical performance of 3d metal oxide.^29–32^

The electrospinning method has received much attention in recent decades as a simple, low-cost, and scalable technique for preparing nanostructured functional materials. The high-voltage electric field exerted on polymer solution not only facilitates solvent evaporation but also induces the polymer to assemble into a fibrous morphology.^33–35^ When functional substances are dispersed into the polymer solution, nanofibrous composites with hierarchical architectures and unique properties can be obtained by the electrospinning method. Among various 3d transition metal oxides, cobalt-based oxides deserve some concerns in terms of their high lithium storage capacity. Owing to its high theoretical capacity,^36–38^ Co_3_O_4_ has always been, by far, the object of electrochemical energy storage field to explore its suitability as an electrode material. The characteristic performance of CoO as an anode material for LIBs through conversion reaction has been rarely investigated. The value of theoretical capacity that CoO can provide is deduced to be comparable to Co_3_O_4_ anode, because of their similar conversion reaction equations and the same Co redox center.^39,40^ Naturally, the electrochemical performance of CoO is largely related to its architectural structure. Designing nanostructured CoO/conductive carbon composite is one of the most effective strategies to achieve its theoretical capacity.^41^ In this paper, Co(NO_3_)_2_/PVP nanofibrous precursors were synthesized by the electrospinning method, and CoO/C nanofibrous composites with ultrafine CoO nanocrystals embedded in carbon matrix were obtained after high-temperature calcination. The resulting CoO/C composite nanofibers exhibited excellent electrochemical properties when evaluated as an anode material for LIBs.

## 2. Experimental section

### 2.1. Materials

Cobalt nitrate hexahydrate (Co(NO_3_)_2_·6H_2_O, AR, Sinopharm Group), *N*,*N*-dimethylformamide (DMF, AR, Sinopharm Group), and polyvinylpyrrolidone (PVP, *M*_W_, = 13 000 000, AR, Sinopharm Group) were directly used as received.

### 2.2. Preparation of CoO/C composite nanofibers

**Fig. 1:**
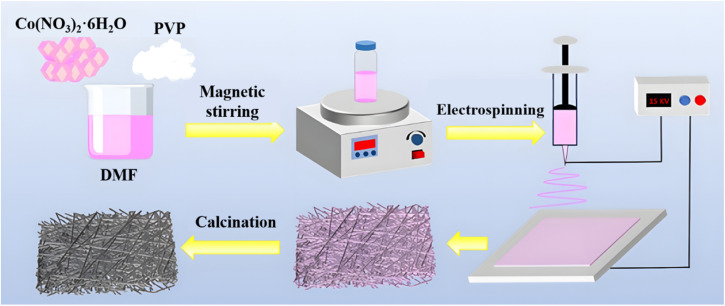
Illustration of preparing CoO/C composite nanofiber.

The CoO/C composite nanofibers were synthesized by a facile electrospinning method followed by high-temperature calcination. Fig. 1 schematically illustrates the synthesis process. 0.7276 g of Co(NO_3_)_2_·6H_2_O was weighted and dissolved in 7 g of DMF, and a certain amount of PVP was subsequently added when Co(NO_3_)_2_·6H_2_O was completely dissolved. A purple transparent solution was obtained after being stirred at 60 °C for 12 h. The viscous purple solution was loaded into a plastic syringe, and the Co(NO_3_)_2_/PVP nanofibrous precursor was obtained by electrospinning. The parameters of electrospinning were set as follows: a DC voltage of 18 kV, a distance of 18 cm between the syringe needle and the aluminum collector, and a feed rate of 0.7 mL h^−1^. The obtained Co(NO_3_)_2_/PVP nanofibrous precursor was placed in a tube furnace and calcined at 450 °C for 3 h in Ar atmosphere, with a heating rate of 5 °C min^−1^. After naturally cooled to room temperature, a black CoO/C powder was obtained. For convenience, the products prepared with 0.6, 0.8, and 1.0 g of PVP were denoted as CoO/LC, CoO/MC, CoO/HC, respectively.

### 2.3. Characterization

The morphological characteristics and microstructures of the CoO/C samples were examined using a field emission scanning electron microscope (SEM, JEOL JSM7500F) and a high-resolution transmission electron microscope (HRTEM, JEOL 2100F). The crystal structures of CoO/C samples were analyzed by an X-ray diffractometry (XRD, D8 Advance, Bruker) with Cu Kα radiation (*λ* = 1.54056 Å) at a tube current of 20 mA and a tube voltage of 40 kV. In addition, the chemical composition and the valence state of the CoO/C samples were analyzed using an X-ray photoelectron spectrometer (XPS, PerkinElmer PHI 1600 ESCA). The carbon content in CoO/C composite nanofiber was determined by thermogravimetric analysis (TGA, TA Instruments, Q5000IR). A laser micro Raman spectrometer (Renishaw inVia, Renishaw, 532 nm excitation wavelength) was used to analyzed the structural properties of the carbon matrix derived from PVP.

### 2.4. Electrochemical measurements

The CoO/C sample and the conductive agent (acetylene black) were firstly mixed well in an agate mortar, then a 10 wt% polyvinylidene fluoride (PVDF) in 1-methyl-2-pyrrolidone (NMP) was added. The mixture solution was continuously mixed until a homogeneous slurry was obtained. The mass ratio of the CoO/C sample, conductive agent, and polyvinylidene fluoride was 75 : 15 : 10. The above viscous slurry was coated on a clean copper foil, and then dried at 120 °C for 12 hours under vacuum. The mass loading of active substance was about 1.9 mg cm^−2^. 2032 two-electrode button cell was assembled in an Unilab-2000 glove box. Lithium metal foil was used as counter electrode. The electrolyte was 1 M LiPF_6_ dissolved into a 1 : 1 (V/V) dimethyl carbonate (DMC)/ethylene carbonate (EC) mixed solvent. The polypropylene microporous membrane (Celgard 2300) served as the separator between the working and counter electrodes. CV measurement was conducted by a PGSTAT 302N (Switzerland) within 0.01–3.0 V at 25 °C. EIS measurement was also conducted by a PGSTAT 302N with an EIS detection frequency from 100 kHz to 10 Hz and a voltage amplitude of 5 mV. Specific capacity, rate capability, and cycling performance were evaluated by a CT2001A battery tester within 0.01–3.0 V at 25 °C. All batteries were allowed to rest for 5 hours before initiating the tests.

## 3. Results and discussion

**Fig. 2:**
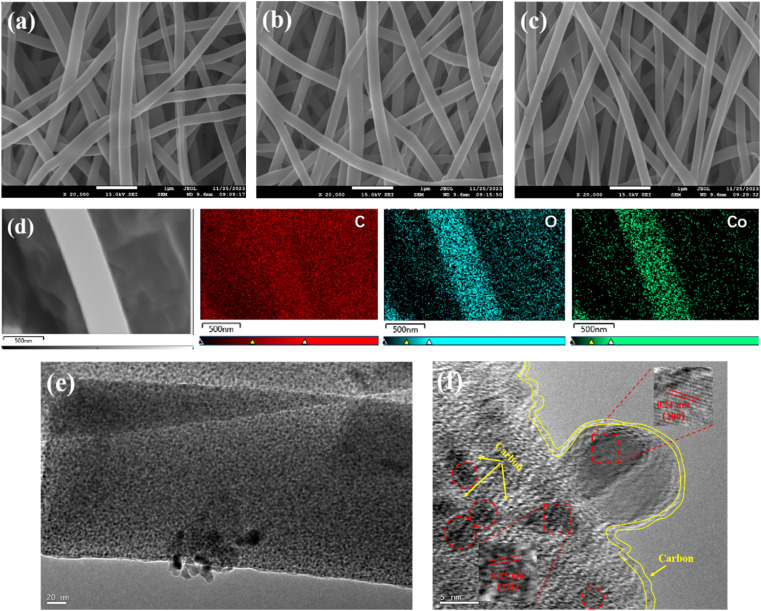
(a) SEM image of CoO/LC; (b) SEM image of CoO/MC; (c) SEM image of CoO/HC; (d) EDS image of CoO/MC and the corresponding elemental mappings of carbon (red), oxygen (blue), manganese (green); (e and f) HRTEM images of CoO/MC.

Fig. 2a–c show the scanning electron microscope (SEM) images of CoO/LC, CoO/MC, and CoO/HC composite nanofibers, respectively. It can be clearly observed that all three samples exhibit high uniformity with a nanofibrous morphology. These fibers are inter-crosslinked with each other, with an average diameter of about 250 nm. Generally speaking, such nanofibrous structures provide a large specific surface area, which can enlarge the contact area between the electrode and the electrolyte, thus improving the rate performance. However, a larger specific surface area often leads to the formation of more substantial solid electrolyte interphase (SEI) film. As displayed in Fig. 2a–c, the surfaces of all CoO/C nanofibers appear smooth and dense, which favors to reduce the initial irreversible capacity loss caused by SEI film. Consequently, these morphological features are beneficial for improving rate performance without exacerbating irreversible lithium loss. Fig. 2d presents the EDS mapping results of CoO/MC, which clearly demonstrate the uniform distribution of C, Co, and O elements throughout the sample, suggesting that the pyrolyzed carbon derived from PVP and the CoO particles are uniformly dispersed with each other. Fig. 2e shows the high-resolution transmission electron microscopy (HRTEM) image of the CoO/MC composite nanofiber, revealing that CoO particles are uniformly dispersed within the nanofibrous composite. As observed in Fig. 2f, primary CoO particles exhibit a roughly spherical shape with an average size of about 5 nm. Additionally, the pyrolyzed carbon derived from PVP is well-dispersed around CoO nanoparticles, indicating that CoO nanoparticles are well encapsulated by the pyrolyzed carbon.

**Fig. 3:**
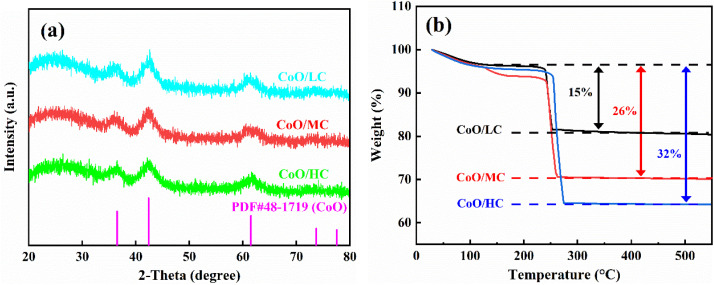
(a) XRD patterns of three CoO/C samples; (b) TG curves of three CoO/C samples.

Fig. 3a presents the X-ray diffraction (XRD) patterns of CoO/LC, CoO/MC, and CoO/HC composite nanofibers. Three samples exhibit identical characteristic diffraction peaks, which correspond perfectly to the diffraction peaks of cubic-phase CoO (PDF no. 48-1719, space group *Fm*3*m*) reported in previous studies.^42^ Specifically, the diffraction peaks at 2*θ* angles of approximately 36.49°, 42.38°, 61.49°, 73.67°, and 77.53° can be indexed to the (111), (200), (220), (311), and (222) crystallographic planes of cubic-phase CoO, respectively. The broad diffraction peak centered at around 25° is visible in Fig. 3a, suggesting the presence of amorphous carbon, which was derived from the pyrolysis of PVP. The broadened and relatively weak diffraction peaks indicate that CoO in all composites exists in a nanoscale form with low crystallinity. Additionally, the peak intensities of the three samples are nearly identical, indicating that varying the amount of PVP does not exert a significant influence on the crystal structure of CoO. Fig. 3b shows the thermogravimetric (TG) curves of three CoO/C samples. The carbon contents of CoO/LC, CoO/MC, and CoO/HC are 15, 26, and 32 wt%, respectively.

**Fig. 4:**
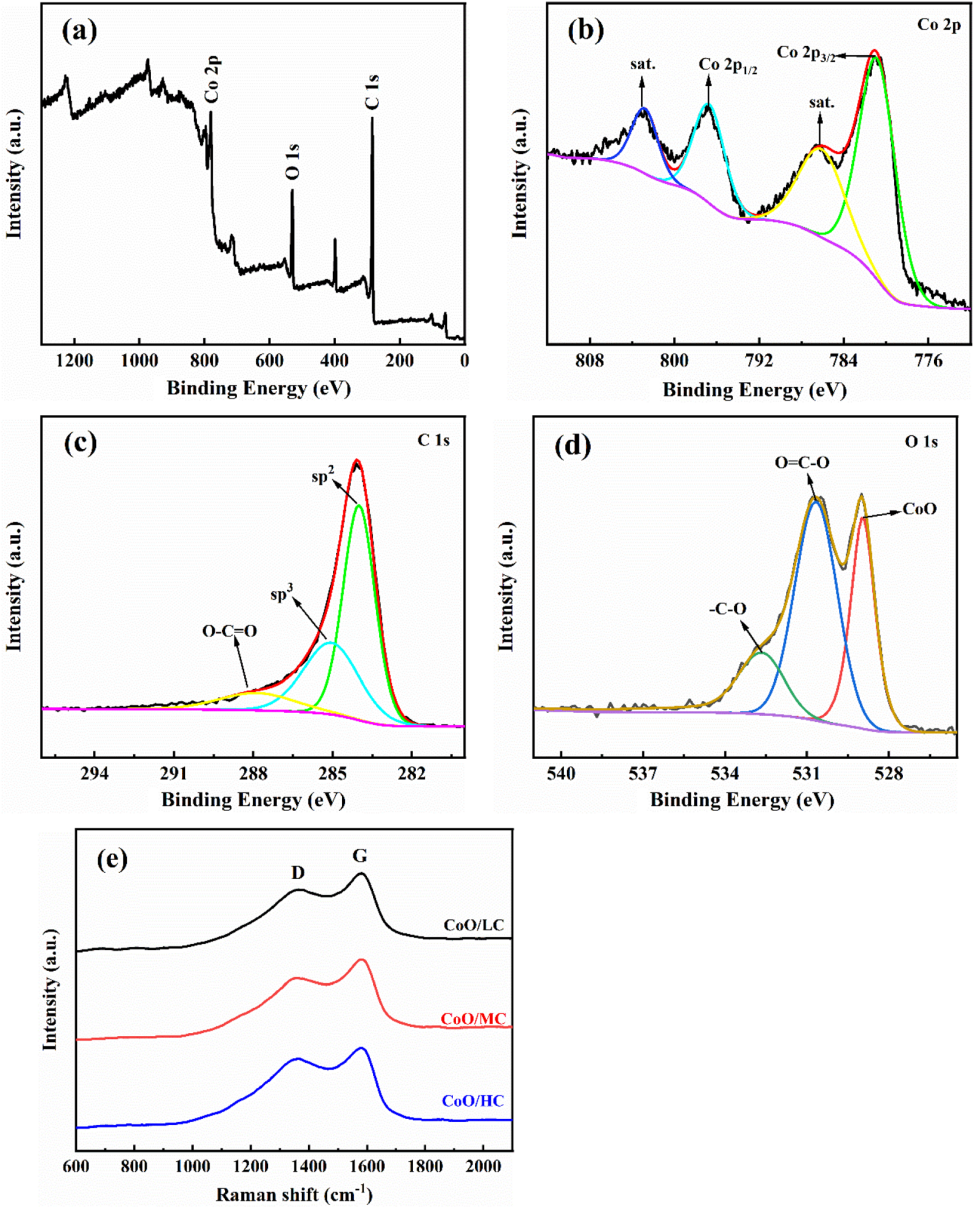
(a) The full XPS spectra of CoO/MC sample; (b) Co 2p spectrum of CoO/MC sample; (c) C 1s spectrum of CoO/MC sample; (d) O 1s spectrum of CoO/MC sample; (e) the Raman spectra of three CoO/C samples.

The structural characteristics of the CoO/MC sample was also investigated using an X-ray photoelectron spectroscopy (XPS). The survey spectrum (Fig. 4a) clearly confirms the presence of carbon (C), oxygen (O), and cobalt (Co) elements. The high-resolution Co 2p XPS spectrum (Fig. 4b) presents two main peaks at 796.30 eV (Co 2p_1/2_) and 780.60 eV (Co 2p_3/2_), which are in good agreement with the characteristic peaks of Co^2+^ in the CoO phase.^43^ Additionally, the characteristic satellite peaks corresponding to Co 2p_1/2_ and Co 2p_3/2_ are observed at 803.21 eV and 786.50 eV, further confirming the localized 3d^7^ electronic configuration of Co^2+^. The high-resolution C 1s spectrum (Fig. 4c) can be deconvoluted into three distinct peaks. Among them, two relatively strong peaks at 284.09 eV and 285.09 eV are associated with sp^2^ C and sp^3^ C, respectively, and one weaker peak at 287.85 eV are associated with O–C=O. The O 1s XPS spectra is shown in Fig. 4d, and three peaks at 529.2 eV, 530.9 eV, and 532.7 eV correspond to the oxygen species in CoO phase, O=C–O groups, and –C–O groups, respectively.^44,45^ The structural properties of the carbon matrix derived from PVP were characterized by Raman spectroscopy. As shown in Fig. 4e, there are two peaks at approximately 1356 cm^−1^and 1580 cm^−1^, corresponding to the D and G bands, respectively. The G band generally represents sp^2^ hybridized graphitic carbon, while the D band represents sp^3^ hybridized disordered carbon. The *I*_D_/*I*_G_ ratios of CoO/LC, CoO/MC, and CoO/HC is sequentially 0.837, 0.819, and 0.843, suggesting that CoO/MC would have a better electronic conductivity compared with CoO/LC and CoO/HC.

**Fig. 5:**
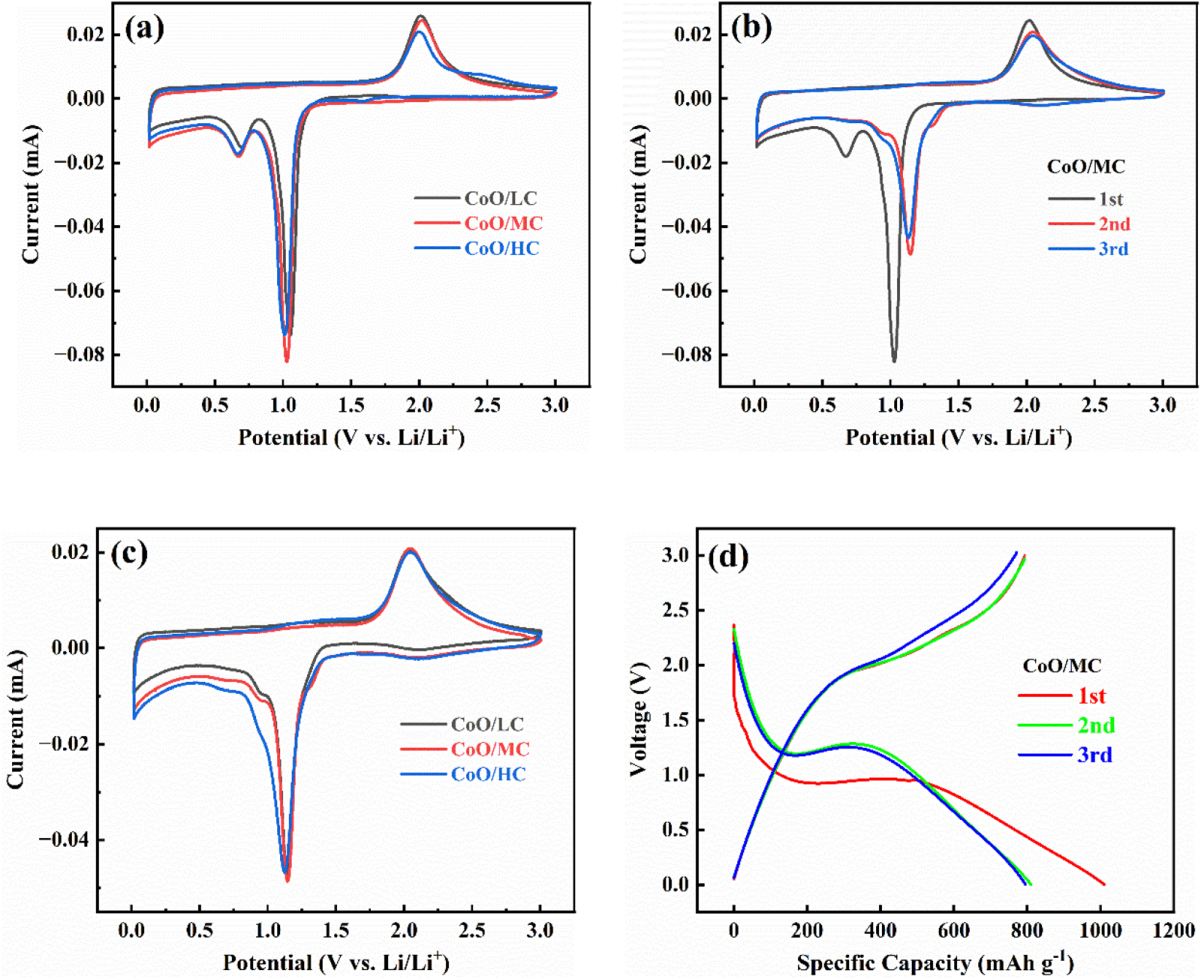
(a) The first CV profiles for CoO/LC, CoO/MC, CoO/HC electrodes at 0.1 mV s^−1^. (b) Evolution of CVs obtained by cycling CoO/MC electrode at 0.1 mV s^−1^. (c) The third CV profiles for CoO/LC, CoO/MC, CoO/HC electrodes at 0.1 mV s^−1^. (d) Evolution of galvanostatic charge/discharge profiles obtained by cycling CoO/MC electrode at 100 mA g^−1^.

The electrochemical behaviors of CoO/C samples were detected by CV tests and galvanostatic charge/discharge tests. The CV scanning rate was 0.1 mV s^−1^, with a voltage range of 0.01–3.0 V. Fig. 5a compares the first CV plots for the three electrodes, which exhibit similar anodic/cathodic behaviors. A pair of distinct redox peaks is observed at 1.03 V (reduction) and 2.02 V (oxidation), corresponding to the reduction of CoO (accompanied by the formation of Li_2_O) and the oxidation of Co (accompanied by the decomposition of Li_2_O), respectively. Additionally, another relatively weak reduction peak is observed at 0.67 V, which is ascribed to the formation of the solid electrolyte interphase (SEI) film. Among the three electrodes, CoO/MC displays the highest redox peak, indicating that it possesses the fastest electrode reaction kinetics.

Fig. 5b presents the initial three CV plots for the CoO/MC electrode. Notably, the second CV plot differs distinctly from the first one. Specifically, the reduction peak observed at 1.03 V in the first cycle undergoes a distinct positive shift to 1.14 V in the second cycle, accompanied by a significant decrease in peak intensity. While the oxidation peak shifts only slightly from 2.02 V to 2.04 V, accompanied by an obvious decrease in peak intensity. This phenomenon of redox peaks shifting to higher potentials may be caused by the amorphous Li_2_O, Co, and CoO phases formed during the initial cycle.^46^ Furthermore, the SEI-related reduction peak below 0.8 V disappears almost entirely in the second cycle, indicating that a complete and stable SEI film has formed on the surface of CoO/MC electrode during the first cycle. This phenomenon may be associated with the unique morphological characteristics of CoO/MC: its smooth and dense nanofiber surface enables the formation of a complete and stable SEI film during the first cycle. Consequently, in the subsequent CV cycles, the reduction peak related to the formation of the SEI film disappears, and the irreversible lithium consumption caused by the formation of the SEI film has also ceased, which will significantly increase the coulombic efficiency in the subsequent CV cycles. The third CV curve almost coincides with the second one, indicating that the CoO/MC electrode activated after the first cycle demonstrates excellent electrochemical stability and reversibility. A comparative analysis of the third CV cycles for three CoO/C electrodes (Fig. 5c) reveals remarkably similar electrochemical characteristics, with all electrodes exhibiting a single pair of redox peaks centered at ∼2.04 V (oxidation) and ∼1.14 V (reduction). Notably, the CoO/MC electrode still maintains the highest peak intensities among three electrodes in the third CV cycle, further indicating that CoO/MC possesses the fastest electrode process kinetics. The electrochemical reaction mechanism of CoO/C electrode follows the reversible conversion: CoO + 2Li^+^ + 2e^−^ ↔ Co + Li_2_O.

Fig. 5d presents the initial three charge/discharge profiles for the CoO/MC electrode measured between 0.01–3.0 V at 100 mA g^−1^. In the first discharge process, a prolonged voltage plateau is observed between 1.07–0.95 V, followed by a voltage slope from 0.95 V down to 0.01 V. According to the CV curves showed in Fig. 5b, this prolonged discharge voltage plateau is associated with the reduction of CoO to metallic Co. While the voltage slope is primarily associated with the formation of the SEI film, coupled with the intercalation of Li^+^ into the amorphous carbon matrix. The voltage plateau shifts to a higher potential range (1.28–1.11 V) in the second discharge process, which is primarily ascribed to the phase transformation of CoO from a crystalline to an amorphous form. The first charge curve exhibits a voltage slope between 2.0–3.0 V. Notably, the second charge curve coincide closely with the first one. These electrochemical evolutions observed in the charge/discharge curves for CoO/MC are consistent with those depicted in its CV plots (Fig. 5b). Additionally, the third charge/discharge curves nearly overlap with the second ones, thereby confirming the excellent electrochemical stability and reversibility of the CoO/MC electrode.

**Fig. 6:**
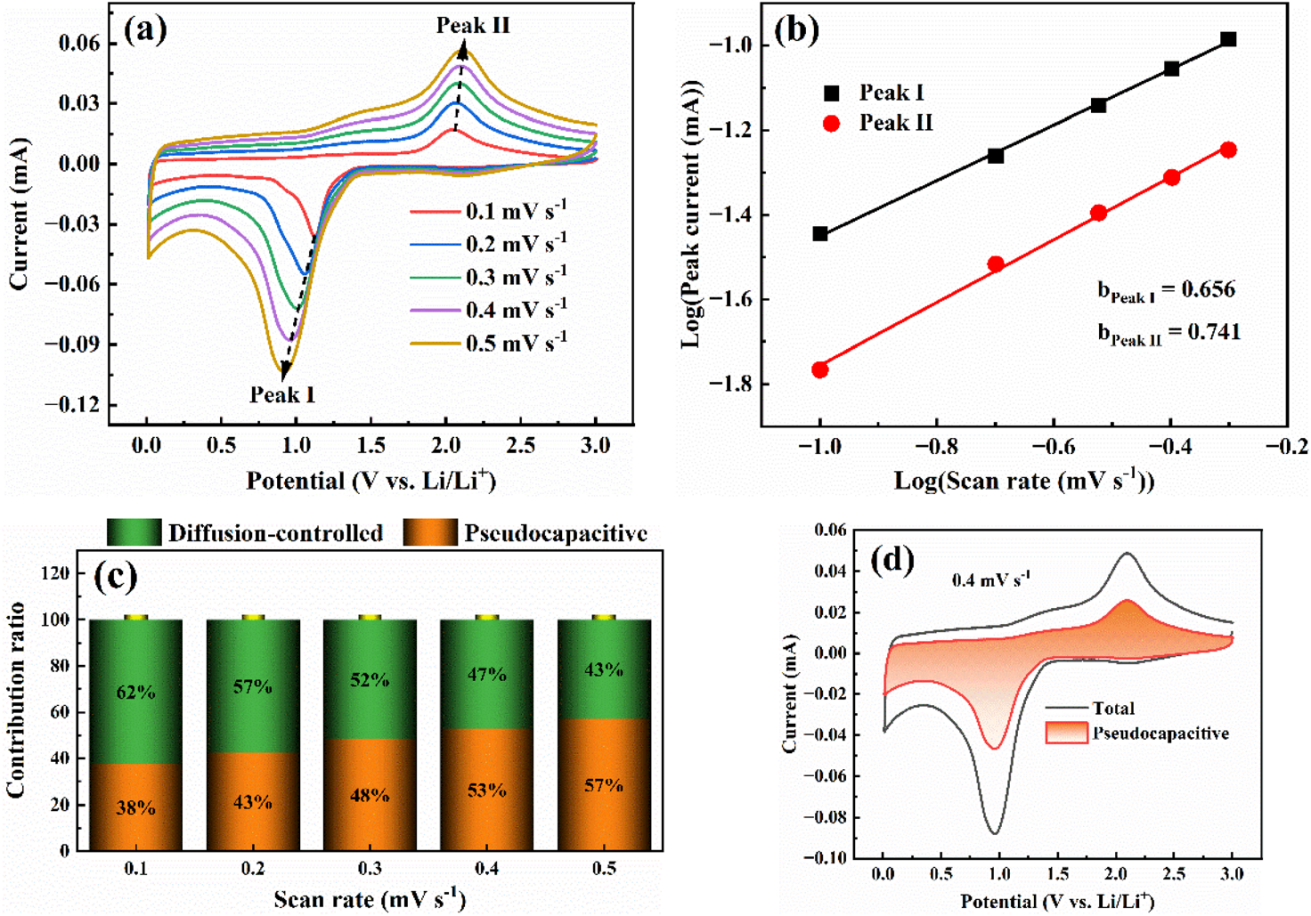
(a) CV plots of CoO/MC electrode at different scanning rates; (b) log *i vs.* log *v* plot for CoO/MC electrode; (c) contribution ratio of the capacitance-controlled and diffusion-controlled charge *versus* scanning rate; (d) Separation of the capacitive and diffusion currents in CoO/MC at a scanning rate of 0.4 mV s^−1^.

To investigate the capacity origin of CoO/MC, its CV curves measured at different scanning rates are showed in Fig. 6a. To minimize the interference from the formation of SEI film, the CoO/MC electrode was firstly cycled twice at 0.1 mV s^−1^. As the scanning rate increases from 0.1 mV s^−1^ to 0.5 mV s^−1^, the peak shape remains almost unchanged, indicating that this electrode's redox reactions possess excellent electrochemical reversibility. It is well established that the capacity of anode materials mainly originates from two processes: the capacitance-controlled process and the diffusion-controlled process. The contribution rate of two processes can be quantified using the following equations:

1 *i* = *av*^*b*^

2 *i* = *k*_1_*v* + *k*_2_*v*^1/2^

where *i* and *v* denotes the measured current and the CV scanning rate, respectively. And *a* and *b* are two variables, where the *b*-value reflects the relative contribution of the two processes. Specifically, the *b*-value near 0.5 indicates that the electrode reaction is dominated by the diffusion-controlled process, while the *b*-value near 1.0 signifies a capacitance-controlled processes. When the *b*-value is between 0.5 and 1.0, the reaction is co-governed by the capacitance-controlled process and the diffusion-controlled process. The *b*-value can be calculated from the linear fitting plot of log *i versus* log *v*. As shown in Fig. 6b, the *b*-values for peak I and peak II are calculated to be 0.656 and 0.741, respectively, demonstrating that the electrode kinetics of CoO/MC are co-governed by the capacitance-controlled process and diffusion-controlled process. The capacitive contribution rates are calculated to be 38%, 43%, 48%, 53%, and 57% at scanning rates of 0.1, 0.2, 0.3, 0.4, and 0.5 mV s^−1^, respectively (Fig. 6c). At the low scanning rate of 0.1 mV s^−1^, the capacitive contribution is relatively low (38%), suggesting that lithium storage is primarily governed by the diffusion-controlled process. But the capacitance-controlled process gradually dominates with the increase of scanning rate, reaching 57% at 0.5 mV s^−1^. This is mainly because ultrafine CoO nanocrystals can shorten the migration distance of Li^+^ ions, while the carbon matrix can enhance electron transport and suppress volume expansion during the repeated insertion/extraction of Li^+^ ions. Pseudocapacitive charge storage can alleviate the kinetic barriers caused by solid-state diffusion processes and rapid charge transfer, thereby favoring the improvement of the rate performance of CoO/MC electrodes.^47^ As a representative illustration, the separation of capacitance-controlled and diffusion-controlled currents for CoO/MC at a scanning rate of 0.4 mV s^−1^ is presented in Fig. 6d.

**Fig. 7:**
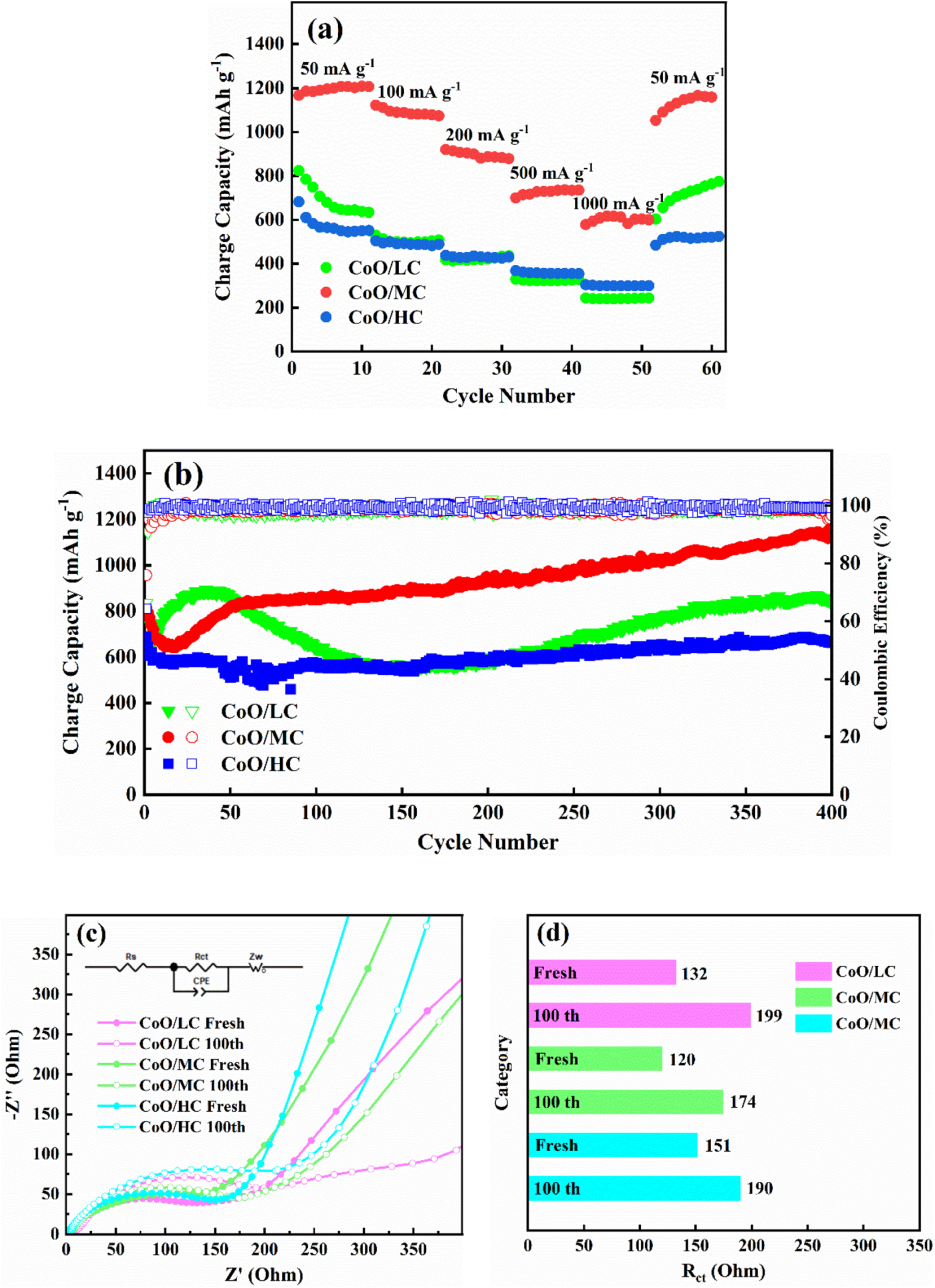
(a) Rate performances of CoO/LC, CoO/MC, CoO/HC. (b) Cyclic performance of CoO/LC, CoO/MC, CoO/HC at 100 mA g^−1^. (c) The EIS spectra for the fresh and cycled electrodes of CoO/LC, CoO/MC, CoO/HC. (d) The corresponding *R*_ct_ values for the fresh and cycled electrodes of CoO/LC, CoO/MC, CoO/HC.

Fig. 7a demonstrates the rate performance for CoO/LC, CoO/MC, and CoO/HC between 0.01–3.0 V. Before the rate performance’ tests, three electrodes were firstly cycled 100 times at 100 mA g^−1^ to activate the electrodes. Among the three samples, CoO/MC exhibits superior rate performance, delivering charge capacities of 1167.6, 1122.0, 919.9, 699.1, and 578.7 mA h g^−1^ at current densities of 50, 100, 200, 500, and 1000 mA g^−1^, respectively. Its charge capacity at 1000 mA g^−1^ reaches 49.6% of that at 100 mA g^−1^. CoO/LC exhibits relatively lower charge capacities of 823.6, 529.6, 417.2, 329.6, and 243.7 mA h g^−1^ at the same current densities. Although CoO/HC exhibits the lowest charge capacities of 681.3, 504.6 at current densities of 50, and 100 mA g^−1^, its charge capacity reaches 437.8, 367.9, and 303.7 mA h g^−1^ at current densities of 200, 500 and 1000 mA g^−1^, outperforming CoO/LC. Remarkably, when the current density is reduced back to 50 mA g^−1^ after cycling at various rates, the charge capacities of CoO/LC, CoO/MC, and CoO/HC recover to 602.4, 1052.6, and 484.5 mA h g^−1^, respectively. Fig. 7b compares the cycling performance of the three samples at a current density of 100 mA g^−1^. The initial charge capacities of CoO/LC, CoO/MC, and CoO/HC are 775.0, 815.3, and 687.7 mA h g^−1^, respectively. After 400 cycles, their charge capacities retain at 843.5, 1147, and 663.9 mA h g^−1^, which are significantly higher than graphite's theoretical capacity. Similar to some transition metal oxides, three CoO/C electrodes exhibit a gradual increasing trend in capacity during deep cycling, which may be related to activation process caused by changes in the crystallinity and structure of CoO.^53–55^ These outstanding cycling performances clearly demonstrate the structural stabilities of CoO/C electrodes are maintained in repeated lithiation/delithiation processes. Table 1 compares the electrochemical performance of CoO/MC with other cobalt-containing anodes. The lithium storage of CoO/MC is impressive among these reported CoO-based anodes. Since PVP is difficult to convert into highly graphitized carbon at 450 °C, the electronic conductivity of the pyrolytic carbon in all CoO/C composite nanofibers is lower than that of highly conductive carbons such as graphene and carbon nanotube. If graphene and/or carbon nanotube are introduced into the CoO/C composite nanofibers, their lithium storage performance will be further improved.

**Table 1:** Comparison of the electrochemical performance of cobalt-containing anodes

Sample	Current density (mA g^−1^)	Cycle number	Charge capacity (mAh g^−1^)	Ref.
CoO/MC	100	400	1147	This work
CoO/C polyhedra	100	50	510	48
CoO/graphene	50	50	650	49
CoO/3D graphene	50	100	860	50
Co_3_O_4_/MWCNT	100	50	860	51
CNTs@CoO@PC	200	300	1090	41
HPC@Co_3_O_4_	200	200	1084	52

The excellent electrochemical performance of these CoO/C composite nanofibers can be primarily attributed to the inherent lithium storage capability of CoO. CoO should have a theoretical capacity comparable to that of high-capacity Co_3_O_4_ anode, owing to their analogous conversion reaction mechanisms involving the same cobalt redox center. More importantly, their superior electrochemical performance is also related to a reasonable synthesis strategy. For the synthesis of CoO/C composite nanofibers, Co(NO_3_)_2_ and PVP were thoroughly mixed in DMF to ensure the uniform dispersion of cobalt and carbon sources. In the electrospinning process, the high-voltage electric field exerted on the mixture solution promoted the instantaneous evaporation of DMF and induced Co(NO_3_)_2_ and PVP to assemble into nanofiber precursors with uniformly mixed components. In the calcination process, Co(NO_3_)_2_ decomposed to form CoO crystals, while PVP was simultaneously transformed into a carbon matrix. The uniform mixing of Co(NO_3_)_2_ and PVP enabled the *in situ* generated carbon to effectively encapsulate the evolving CoO crystallites, thereby inhibiting CoO growth and obtaining ultrafine CoO nanocrystals uniformly dispersed in the carbon matrix. The nanofibrous secondary structure of these composites enlarges the contact area between the electrolyte and the electrode, promoting the rapid transport of ions and electrons. The ultrafine CoO nanocrystals shorten the Li^+^ migration distance, thereby improving the ionic conductivity of the electrode and enabling more CoO species to participate in electrochemical reactions. Additionally, ultrafine CoO nanocrystals are perfectly encapsulated by pyrolyzed carbon, which helps to accelerate electron transfer between CoO and current collector, protect CoO from direct contact with electrolyte, prevent harmful side reactions between electrolyte and CoO, and effectively alleviates the huge volume change of CoO in repeated lithiation/delithiation processes.

Fig. 7c shows the electrochemical impedance spectroscopy (EIS) curves for fresh Co/C electrodes and Co/C electrodes cycled at 100 mA g^−1^ for 100 cycles. All Nyquist plots comprise a semicircle in the high-frequency region and an inclined line in the low-frequency region. A corresponding equivalent circuit is inset in Fig. 7c, where *R*_s_ and *R*_ct_ stand for electrolyte resistance and charge transfer resistance, while *Z*_ω_ and CPE represent Warburg resistance and the constant phase element. The *R*_ct_ values derived from the EIS analysis are summarized in Fig. 7d. Among the three fresh electrodes, the CoO/MC electrode exhibits the lowest charge transfer resistance, with a value of 120 Ω. After 100 cycles, all electrodes show a moderate increase in *R*_ct_ value. The CoO/MC electrode still maintains the lowest charge transfer resistance of 174 Ω among the cycled samples. This result further confirms that CoO/MC possesses the optimal electrochemical performance.

## 4. Conclusions

A simple and effective electrospinning method and subsequent calcination was used to successfully synthesize CoO/C composite nanofibers. XRD analysis confirmed that the composites consisted of cubic-phase CoO and amorphous carbon. SEM analysis showed that the CoO/C composites exhibited a well-defined nanofibrous morphology with a diameter of about 250 nm. HRTEM analysis confirmed that the primary CoO crystallites (∼5 nm) were perfectly encapsulated within an amorphous carbon derived from PVP pyrolysis. CVs at various scan rates revealed that the electrode kinetics of CoO/MC were co-governed by the capacitance-controlled process and diffusion-controlled process. The capacitive contribution rates for CoO/MC were calculated to be 38%, 43%, 48%, 53%, and 57% at scanning rates of at scanning rates of 0.1, 0.2, 0.3, 0.4, and 0.5 mV s^−1^, respectively. When evaluated as an anode material for LIBs, CoO/C composite nanofibers exhibited exceptional cycling stability and rate capability. Notably, at a current density of 100 mA g^−1^, CoO/MC delivered a first charge capacity of 815.3 mA h g^−1^ and maintained an exceptional charge capacity of 1147 mA h g^−1^ after 400 cycles. At a high current density of 1000 mA g^−1^, its charge capacity reached 49.6% of that at 100 mA g^−1^. The electrospinning method is a simple, low-cost, and scalable technique for preparing nanostructured functional materials. Although the cost of cobalt raw materials is high, the simple production processes and the low energy consumption can mitigate some of the cost concerns the cost of Co resources. In addition, the recycling of cobalt-based anode materials is more convenient and safer than that of graphite anodes. Therefore, the CoO/C composite nanofibers emerge as a promising anode material for high-performance LIBs.

## Author contributions

Zonghui Yi designed the experiments, interpreted the results and wrote the manuscript. Zhiyuan Cheng completed the preparation and characterization of materials, and participated in the writing of the paper. Hui Zhang, Lin Xie, and Hai e Yang participated in the testing of materials.

## Conflicts of interest

There are no conflicts to declare.

## Data Availability

All data supporting the findings of this study are included within the article. Additional raw data, characterization files, and experimental details are available from the corresponding author upon reasonable request.
